# Progress Toward Development of Effective and Safe African Swine Fever Virus Vaccines

**DOI:** 10.3389/fvets.2020.00084

**Published:** 2020-02-21

**Authors:** Huldah Sang, Gabrielle Miller, Shehnaz Lokhandwala, Neha Sangewar, Suryakant D. Waghela, Richard P. Bishop, Waithaka Mwangi

**Affiliations:** ^1^Department of Diagnostic Medicine/Pathobiology, Kansas State University, Manhattan, KS, United States; ^2^Department of Veterinary Pathobiology, Texas A&M University, College Station, TX, United States; ^3^Department of Veterinary Microbiology and Pathology, Washington State University, Pullman, WA, United States

**Keywords:** ASF, vaccine, attenuated virus, subunit vaccine, live vector

## Abstract

African swine fever is a major concern due to its negative impact on pork production in affected regions. Due to lack of treatment and a safe vaccine, it has been extremely difficult to control this devastating disease. The mechanisms of virus entry, replication within the host cells, immune evasion mechanisms, correlates of protection, and antigens that are effective at inducing host immune response, are now gradually being identified. This information is required for rational design of novel disease control strategies. Pigs which recover from infection with less virulent ASFV isolates can be protected from challenge with related virulent isolates. This strongly indicates that an effective vaccine against ASFV could be developed. Nonetheless, it is clear that effective immunity depends on both antibody and cellular immune responses. This review paper summarizes the key studies that have evaluated three major approaches for development of African Swine Fever virus vaccines. Recent immunization strategies have involved development and *in vivo* evaluation of live attenuated virus, and recombinant protein- and DNA-based and virus-vectored subunit vaccine candidates. The limitations of challenge models for evaluating ASFV vaccine candidates are also discussed.

## Introduction

African swine fever is caused by a DNA virus classified in the *Asfarviridae* family, genus *Asfivirus* (1). The pathogen is an arthropod-borne highly complex enveloped double-stranded DNA virus which primarily replicates in the host cell cytoplasm (2, 3). The virus is easily transmitted since it is extremely stable and persists under a variety of environmental conditions, for up to several months, thus creating a requirement for implementation of strict biosecurity measures to prevent transmission (4). The virus causes a highly contagious hemorrhagic disease in pigs that produces a wide spectrum of clinical syndromes ranging from rapid lethality to relatively mild symptoms. The internal lesions closely resemble those of the unrelated classical swine fever virus but with higher morbidity and mortality rates (5). ASF is an economically important disease that is currently enzootic in sub-Saharan Africa (24 genotypes described based on the sequence of the c-terminus of the p72 surface antigen) and Sardinia (p72 genotype 1). In 2007 a genotype II virus from Southeast Africa reached the Caucasus region and subsequently Russia and Eastern Europe (6, 7). Multiple outbreaks almost certainly originating from the single index case in the Caucasus have recently (from August 2018) been reported in China, Vietnam, Cambodia, Laos, North and South Korea, Philippines, and Timor-Leste (OIE, December 2019). The consequences for the 450 million pigs in China are already devastating. Given the level of global interconnectivity of the world economy and the stability of the virus, there is a high risk of spread to ASFV-free large scale pork producing countries, such as U.S.A, Germany, Denmark, and Brazil (7).

As the causal agent of one of the most severe diseases of domestic pigs that spreads easily, in the case of the major genotype II pandemic facilitated by the movement of wild boar in which the disease is lethal, ASFV has many sanitary and socio-economic consequences which significantly impact the national and international trade of animals and animal products (8). At present, mass slaughter of infected and in-contact pigs with proper disposal and disinfection is the only way to manage outbreaks. The host cell entry and replication mechanisms utilized by the virus, the strategies it uses to evade host defense systems, identity of viral proteins that are important in causing an effective host immune response, and the protective immune mechanisms involved, are gradually being discovered (9). Since completion of sequencing of the first entire virus genome (10), a concerted effort has been made to analyze the genomes and predicted proteome of multiple isolates to generate knowledge that is vital for designing innovative disease control strategies, which include an effective vaccine against various ASFV genotypes (11–14).

Attempts to develop a safe vaccine for protection of pigs against ASFV have continued without significant success from the time ASFV was first isolated (15). Without a safe and efficacious vaccine, pig farmers in the affected areas are venerable to the disease whose prevention depends exclusively on ensuring that infected pigs, contaminated feeds and materials, or fomites (for example virus on the clothes or shoes of pig workers) are not introduced into areas that are ASFV-free (16). All eradication programs that have proven successful involved the prompt diagnosis, quarantine, slaughter, and properly discarding all animals in infected sites (17–19). Subsequently, surveillance of all pig farms within a specific region must be conducted to ensure maintenance of disease-free zones.

The focus of this review is the historical progress made so far in regards to the efforts directed at development of safe and effective vaccines for protection of swine against ASF virus. Several prospective vaccine candidates have been evaluated and some novel candidates are being developed and tested. The development strategies for the vaccine can be divided basically into these broad categories; live attenuated ASF viruses, inactivated ASF virus, live-vectored subunit, mammalian expression plasmid DNA-based, recombinant protein-based-subunit candidates, and a combination of the above (20). Live attenuated virus can be generated by deletion of genes encoding virulent factors for safe induction of protective immunity (21, 22). Some ASFV antigens have been identified and used to generate recombinant proteins for evaluation of protein-based candidate immunogens (23). Direct delivery of viral nucleic material into host cells can result in *de novo* gene expression and the expressed antigen can elicit immune responses. Live-vectored vaccines are similar to nucleic acid-based vaccines except that the genes encoding target antigens are delivered into the host cell by employing non-pathogenic attenuated virus or bacteria. There are constraints to all of these approaches that have prevented rapid progress in development of safe and cost effective vaccines to control the virus.

## Live Attenuated ASFV Vaccine Candidates

A range of mutant viruses have been either isolated from the field or experimentally generated and tested for their ability to safely induce protective immunity in pigs and wild boars. Attenuated viruses can be either naturally occurring low-virulence isolates or virulent strains attenuated by deletion of defined DNA sequences encoding virulence factors. Whole virus-based vaccines can be sub-divided into two categories: live attenuated viruses and inactivated or killed viruses.

### Live Attenuated Vaccine Candidates

Live attenuated ASFV vaccine candidates can induce protective immunity, but the use of naturally attenuated strains of ASFV has the potential to cause post-vaccination reactions and side effects. Although it has previously been demonstrated that following subclinical infections of domestic pigs with low virulent strains of ASFV, immunity against homologous, but not heterologous, challenge was conferred (24). A Portuguese group was the first to demonstrate subclinical infections of domestic pigs with low virulent strains of ASFV (20). They found that pigs immunized with the naturally occurring ASFV NH/P68 virus, which was isolated subsequent to the introduction of a genotype I virus into that country from Angola, were protected against challenge with virulent ASFV L60 and this correlated with increased NK cell activity (20). Immunization of pigs with low virulence ASFV isolates provide varying levels of protection against challenge with virulent virus. For instance, pigs immunized with naturally attenuated ASFV strains NH/P68 or the *Ornithodoros erraticus* tick-derived OURT88/3 were protected following challenge with closely related ASFV strains and those challenged with heterologous strains were partially protected (20, 25–27). The level of protection in both cases varied from 60 to 100% (26–32). These outcomes provided useful data concerning immune parameters involved in protection. Both antibodies and cytotoxic CD8^+^ T cells were demonstrated to play important roles in conferring protection (25, 33–35).

Despite the ability to induce protective antibody and T cell responses, naturally attenuated isolates have been associated with adverse side effects and safety concerns (29). To improve safety, mutant viruses have been generated with deletions of genes involved in virulence and progress of clinical disease (DP96R and DP71L) and inhibition of IFN-γ (A276R) (23, 36). However, varying levels of protection were observed in immunized pigs. Virulent virus isolates can be attenuated by deletion of rationally selected genes encoding virulence factors to obtain attenuated virus that can safely induce protective immunity. However, deletion of some genes has been shown to significantly reduce the virulence of the virus in pigs, whereas deletion of others had no apparent effect (37). In one study, deletion of virulence genes DP96R and DP71L from the ASFV OURT88/3 isolate reduced its ability to protect against challenge with virulent virus OURT88/1 isolate, whereas in another study, 60–100% protection was observed following challenge with heterologous virulent ASFV Armenia 07 (23, 29). It has been shown that deletion of IFN-γ inhibitor genes DP148R, MGF360, and 530/505 genes from ASFV Benin97/1 isolate induced protective immune responses against challenge (38, 39). By contrast, deletion of the early virus protein L83L from the ASFV Georgia 2007 isolate did not reduce viral virulence in experimentally infected swine, and no challenge studies were performed (40). Recently, immunization of pigs with a naturally attenuated genotype II ASFV Lv17/WB/Rie1 isolated from wild boars in Latvia conferred protection upon challenge through contact with animals infected with virulent ASFV (41).

Immunization with attenuated virus, rather than with selected antigens, is advantageous since it elicits immune responses against all the viral antigens that are normally encountered by the host during the course of an infection, and it may therefore be more effective. Several attenuated viruses have been tested for their ability to induce immune protection (Table 1). Among the genes that have been deleted in these attenuated viruses are; EP402R (a homolog of CD2), B119L, DP71L, K169R, DP96R, E165R, EP153R, MGF360/530, A224L, A238L, and E269R (46). Many of the proteins encoded by the deleted or inactivated genes in these attenuated constructs have predicted functions based on sequence identity, and biological observations. The product encoded by EP402R is involved in mediating hemadsorption of RBCs to infected host macrophages and extracellular virus particles; DP71L exhibits similarity to a Herpes simplex virus (HSV) neurovirulence factor; KI69R encodes Thymidine kinase; E165R encodes a dUTPase; EP153R encodes a C-type lectin; A22L is an IAP apoptosis inhibitor that presumably prevents host programmed cell death; A238L is an inhibitor of host cell transcription; and E296R encodes an AP endonuclease Class II (47). The function of the MGFs, including families 360 and 530 is unknown, although some of the proteins contain predicted signal peptides, suggesting secretion and interaction with host proteins (47). B119L has sequence identity to several yeast proteins including ERV1 which functions in oxidative phosphorylation (4).

**Table 1:** Live attenuated ASFV vaccines.

**Strain**	**Vaccine virus**	**Protection**	**References**
Naturally attenuated OURT88/3	OURT88/3	Homologous OURT88/3 strain	(23)
		Heterologous OURT88/1 strain	(28)
		Heterologous Benin 97/1, Uganda 65 strains	(28)
NH/P68	NH/P68	Heterologous L60, Armenia 07 strains	(20, 32)
Gene-deletion OURT/88/3	OURT/88/3ΔDP71L	Homologous OURT/88/1strain	(23)
	ΔDP96R		
NH/p68	NH/P68ΔA238L	Homologous L60 strain	(32)
		Heterologous Armenia 07 strain	(32)
	NH/P68ΔEP153R	Homologous L60 strain	(32)
	NH/P68ΔA224L	Homologous L60 strain	(32)
		Heterologous Armenia 07 strain	(32)
Benin97/1	Benin 97/1ΔMGF	Homologous Benin 97/1 strain	(38)
	Benin 97/1ΔDP148R	Homologous Benin 97/1 strain	(39)
Georgia 07/1	Georgia 07/1Δ9G L	Homologous Georgia 07/1 strain	(42)
	Georgia 07/1ΔMGF	Homologous Georgia 07/1 strain	(43)
	Georgia 07/1Δ9GL	Homologous Georgia 07/1 strain	(44)
	ΔDP96R/UK		
	Georgia 07/1ΔB119/	No protection	
	ΔDP71L/ΔDP96R		(45)
Ba71	Ba71ΔEP402R	Heterologous E75 and Georgia 07/1 strains	(30)

Deleting certain genes from the genome of a virulent ASFV isolate affects pathogenesis in pigs (48). For example, when the EP402R gene was deleted, there was reduction in virus dissemination through tissues (49). However, recent studies showed that deletion of the EP402R gene from the genotype I BA71 isolate attenuated the virus and the mutant conferred protection against challenge with homologous virulent BA71 virus, and also heterologous E75 (Genotype 1) and Georgia 2007/1 (Genotype II) viruses (30). Surprisingly, deletion of the DP71L and DP96R genes from the ASFV strain OURT88/3 decreased its protective capacity in pigs following challenge with virulent virus (23). Recent studies have also shown that deletion of the B119L, DP71L/NL, and DP96R/UK genes from the ASFV Georgia 2007/1 strain reduced its replication efficiency, but the mutant did not protect immunized pigs against challenge with parental virus (45).

Deletion of MGF 360, MGF 505, or B119GL genes attenuated the ASFV Georgia 2007/1 isolate and the respective mutant virus elicited immune responses that protected immunized pigs against homologous virulent challenge. However, protection was not observed when both MGF 360/505 and B119GL genes were deleted, indicating that deletion of multiple genes can sometimes significantly reduce protective capacity of the resulting mutant (42, 43, 50). However, by contrast, improved protection and safety was observed when the DP96R/UK and B119GL genes were simultaneously deleted from the ASFV Georgia 2007/1 isolate (44). In the case of other specific virulence genes, such as Thymidine Kinase (TK), although less pathogenic viruses were generated, the performance of the resultant mutants was not consistent. Notably, deletion of the TK gene in Georgia 2007/1 and Malawi strains attenuated the viruses, however the Malawi strain, but not the Georgia 2007/1 strain, induced protective responses in immunized pigs (30, 51, 52). The outcome suggests that the effect of gene deletions on the ability of the virus to elicit immune protection is strain-specific (52). Thus, additional new knowledge is required for rational development of live attenuated ASFV candidate vaccine and that evaluation has to be on a case by case basis.

Although attenuated ASFV is currently the most promising vaccine candidate, there are still major challenges that need to be addressed. These include safety concerns because the viruses are not sufficiently attenuated, requirement for high biocontainment for production of the attenuated virus, availability of suitable cell lines and optimization of culture conditions for vaccine virus scale up which remains a key constraint (53).

### Inactivated ASFV Vaccines

Efforts to generate inactivated or killed ASFV vaccines capable of conferring protection have been unproductive (54–57). One recent study showed that although an inactivated preparation of the ASFV Armenia08 formulated with contemporary adjuvants elicited ASFV specific antibodies, there was no protection upon challenge with homologous virulent virus (11). This outcome raises serious questions regarding the role of antibodies in protection against ASFV, but it is possible that the antibodies elicited by this particular immunogen failed to confer protection. Although antibodies have been implicated in protection against ASFV, the antibody target(s), the actual effector mechanism(s) or the isotype(s) involved, remains unknown (16).

## Subunit Vaccines

Subunit vaccines utilize a defined pathogen structural, non-structural or unassigned proteins as antigens to elicit protective immune responses (58). This is accomplished by using a gene encoding a candidate antigen to generate recombinant antigen that is formulated with an adjuvant. Alternatively, the gene can be used to generate a live-vectored recombinant construct for *in vivo* antigen expression. Several antigens, including p12, p30, p54, and p72, have been evaluated for their protective potential as recombinant proteins. Antibodies against p12 and p72 have been shown to hinder binding of the virus to the host cells, while antibodies against p30 protein prevents the virus from entering cells (37, 46, 48, 59). However, p12-specific antibodies induced in both natural infections and in animals inoculated with inactivated virus or recombinant p12 protein, do not block virus binding to the host cell or neutralize virus infectivity (59).

The p30 and p54 proteins mediate interactions between ASFV and host cells and simultaneous interference with the interactions of these two proteins with the host cells has a complementary effect in antibody-mediated protection (48). Some preliminary vaccination experiments using these recombinant proteins gave promising results and these could be followed up with other combinations of recombinant proteins, either as purified proteins, or recombinant live-vectored virus constructs. For instance, baculovirus-expressed p30 and p54 elicited antibodies that protected pigs against challenge with ASFV E75CV1-4 (48). However, in another study, antibodies elicited against p30, p54, and p72 were not sufficient to confer protection against challenge with the ASFV Pr4 isolate (60). Another study showed that immunization of pigs with baculovirus-expressed EP402R antigen, a viral transmembrane protein, elicited hemadsorption inhibition antibodies and conferred partial protection against lethal challenge (37). Moreover, immunization of pigs with a combination of baculovirus-expressed EP402R and C-type Lectin, induced a significant level of protection following challenge with homologous ASFV (Table 2) (27).

**Table 2:** Protein subunit candidate vaccines.

**ASFV proteins**	**Expression system**	**Protection**	**References**
CD2v	Baculovirus expressed	Partial protection	(37)
p54, p30	Baculovirus expressed	Protection	(48)
p54, p30, p72	Baculovirus expressed	Partial protection	(60)
CD2v and C-type Lectin	Baculovirus expressed	Protection	(27)

## Live-Vectored and Dna-Based Subunit Vaccine Candidates

Gene expression vectors, either viral, bacterial, or plasmid-based have been used as antigen delivery platforms that can be tailored to elicit a desired immune response (Table 3). Only a few studies have been conducted to evaluate immunogenicity and protective efficacy of prototype vectored ASFV subunit vaccine candidates. Argilaguet et al. (49) showed that immunization of pigs with BacMam-sHAPQ, a baculovirus-based construct encoding p30, p54, and secretory hemagglutinin or sHA, induced antigen-specific T-cell responses in pigs. Following challenge, 4/6 of the immunized pigs, but not the negative controls, were free of the virus (49). A recombinant modified vaccinia virus Ankara (MVA) expressing the p72, EP402R, and EP153R antigens, induced T cell responses, but the animals were not challenged to determine whether the induced responses were protective (61). Alphavirus expressing ASFV p30, p54, or p72 were tested for immunogenicity in pigs and the results suggested that an attenuated live virus boost of an initial immunization of a vector-expressed antigen may broaden humoral epitope response (65). It has recently been shown that cocktails of adenoviruses expressing multiple ASFV (Georgia 2007/1) antigens [p32, p54, pp62, p72, A104R, K205R, B438L, EP402RΔPRR, B602L, B119L, and A151R], induced robust cellular and antibody responses (62, 63). Although highly immunogenic, the adenovirus-vectored ASFV antigen cocktail did not confer significant protection following intranasal challenge with ASFV Georgia2007/1 isolate (64), whereas in a sub-study, protection was observed in 5/9 of the vaccinated animals (64). This study further suggested that antibodies induced by one of these adenovirus vectored antigen cocktails may be counter-protective, since delivery using an adjuvant that induced lower levels of antibodies, resulted in enhanced protection of pigs following virus challenge (64). Moreover, recent studies has also shown that a cocktail of Adenovirus and Modified Ankara Virus expressing up to 18 antigens [I215R, I73R, CP530R [pp62], CP204L [p32], MGF110-5L, B646L [p72], MGF110-4L, M448R, L8L, E146L, C129R, A151R, MGF110-1L, L10L, K78R, E184L, E165R, and CP312R] used in a prime-boost strategy induced antigen specific immune responses but failed to protect against challenge (66).

**Table 3:** Live vectored and DNA sub-unit vaccine candidates.

**ASFV proteins/genes**	**Expression system**	**Protection**	**References**
Vectored p54, p30, sHA	BacMam-sHAPQ	Partial protection	(49)
p72, CD2v, and EP153R	Modified vaccinia virus ankara	No challenge study	(61)
7 and 12 antigen cocktails	Adenovirus vectored	No challenge study	(62, 63)
7 antigen cocktail	Adenovirus vectored	Partial protection	(64)
7 antigen cocktail	Adenovirus vectored	No protection	(64)
12 antigen cocktail	Adenovirus vectored	No protection	(64)
p30, p54, and pHA-72	Alphavirus vectored prime,	No challenge study	(65)
	Attenuated OURT88/3 boost		
18 antigen cocktail	Adenovirus and MVA vectored	No protection	(66)
DNA sub-units DNA expression library	DNA constructs	Partial protection	(67)
p54/E183L, p30/CP204L	DNA constructs	No protection	(68, 69)
Ubiquitin-CD2v/pEP402R- p54/E183L-p30/CP204L	DNA constructs	Partial protection	(69)
DNA and vectored/protein 47 antigen pool	DNA constructs and vaccinia virus	Partial protection	(70)
p15, p35, p54, and ±p17 and p32, p72, CD2v, and ±p17	DNA and protein vaccine	No protection	(71)

DNA vaccination involves inoculation of expression plasmid constructs encoding defined target antigens for expression in mammalian host cells. Potential advantages of DNA vaccination over traditional approaches, include stimulation of B-cell, CD4, and CD8 T-cell responses, improved vaccine stability, the absence of any infectious agent and the relative ease of large-scale production, although production to GMP standard may be more expensive than adenovirus (72, 73). A DNA vaccine candidate, pCMV-sHAPQ, encoding ASFV p30 and p54 fused to hemagglutinin extracellular domain (sHA) improved humoral and the cellular responses in pigs, but provided partial protection against lethal challenge with the virulent E75 ASFV-strain (68). Similarly, immunization of pigs with a plasmid construct encoding p30, p54, and sHA genes fused to ubiquitin, elicited T cell responses but conferred partial protection against challenge with lethal E75 virus strain in the absence of neutralizing antibodies. In this study, protection correlated with presence of sHA-specific CD8^+^ T cells (68, 69). A further experiment demonstrated that immunization of pigs with a DNA expression library of more than 4,000 plasmid clones, each one containing a random Sau IIIa restriction fragments derived from the viral genomic DNA fused to ubiquitin conferred 60% protection against lethal challenge with the virulent E75 strain (67).

More recent approaches have evaluated several heterologous prime-boost strategies in an attempt to improve protective efficacy of prototype subunit vaccines. Jancovich et al. (70) showed that pigs primed with DNA plasmids encoding 47 ASFV antigens and boosted with recombinant vaccinia virus expressing the same antigens, significantly reduced ASF viral load in the vaccines following challenge with ASFV Georgia 2007/1. However, the same group showed that immunization of pigs with 12 adenovirus constructs expressing selected ASFV antigens and boosting with vaccinia virus expressing cognate antigens, reduced viral loads but the immunized pigs were not protected against challenge with ASFV OURT88/1 (66). Another study has demonstrated that immunization of pigs with recombinant proteins [p15, p32, p54, and ±p17] and plasmid DNA constructs encoding [p32, p72, EP402R, and ±p17] in a prime and two booster doses induced cell mediated immune responses and antibodies that were shown to neutralize ASFV *in vitro*. However, the immunized pigs were not protected against challenge with Armenia 2007 strain (71).

## Immunization Protocol

The route of vaccine administration is worthy of further research in the context of immunization protocols. For example, it was observed that the naturally tick attenuated genotype I OURT88/3 virus when administered at low to intermediate doses (10^3^–10^4^) pfu was protective against virulent wild type OURT88/1 challenge when administered intranasally, but not when administered intramuscularly at the same doses (74). Most of the ASFV vaccine candidates tested so far have been delivered by parenteral injection. Recent global consortia call for improved effective vaccine delivery systems, amongst others measures, as a roadmap for developing a vaccine (75, 76). An oral bait-based vaccine would be more attractive, particularly for immunization of wild boars and feral pigs. Oral bait-based vaccine delivery has been used for successful immunization of wild animals (77, 78). Notably, a vaccinia virus-vectored rabies vaccine [RABORAL] and an adenovirus-vectored oral bait rabies vaccine [ONRAB] have been used successfully to control rabies in domestic and wild animals in U.S.A and Europe (77, 79, 80). Recently, an oral ASFV vaccine candidate, attenuated genotype II ASFV (Lv17/WB/Rie1), was tested in wild boars and shown to confer 92% protection against virulent challenge with ASFV Armo7 isolate (81). The Lv17/WB/Rie1 mutant has potential to be used for ASFV management in domestic pigs and to control ASFV from spreading in wild boar populations. However, further studies are needed before the vaccine can be approved for deployment.

## Challenge Models and Their Limitations

Lack of knowledge on the appropriate challenge model relevant to the candidate vaccine limits the development of a safe and efficacious ASFV vaccine. Transmission of ASFV in domestic swine often occurs via direct contact between persistently infected and susceptible animals, via soft ticks in the genus *Ornithodoros*, or contaminated feed including other pigs that have been slaughtered or succumbed to the disease (82). ASFV epidemiology is complex since infection of domestic pigs typically results in mortality and morbidity, whereas wild suids including warthogs and bushpigs can be infected but they are asymptomatic. There are also different patterns of pathogenesis and clinical outcomes in domestic pigs across different regions of the world where ASFV is endemic. In addition viral pathogenicity may evolve over time and as the virus expands its range into new areas (1). Genetic variability amongst different breeds of swine, which originate from multiple independent domestication events, could be one factor explaining clinical disease why outcomes vary between different infected animals (1). Factors such as husbandry systems and the involvement of wild boar and tick transmission may also be important. Therefore, simulation of most common natural routes of infection and transmission is critical for evaluation of protective efficacy of vaccine candidates. Currently, live attenuated ASFV are the most promising vaccine candidates for eliciting protective immunity, but safety concerns combined with scale-up issues have delayed progress in deployment of these candidates in the field. The BA71ΔEP402R deletion mutant was shown to protect against lethal challenge with both genotype I strains, BA71 and E75 (30). Additionally, 100% of pigs immunized with the mutant survived lethal challenge with genotype 2 Georgia 2007/1 isolate (30).

The cross protection conferred by BA71ΔEP402R makes this most promising candidate vaccine developed to date. However, biosecurity and biocontainment concerns remain, as well as the requirement to ensure that pigs immunized with this vaccine and others can be differentiated from infected pigs.

Following immunization with candidate vaccines, protection levels vary from 0 to 100%, depending on the breed of pigs, vaccine dose, delivery route, and the virus isolate used for the challenge (30, 63, 64, 70, 81, 83, 84). As mentioned, ideal challenge models should closely resemble natural ASFV transmission in swine and the most common transmission route is likely to be via direct contact through mucosal surfaces (17, 85). Therefore, a novel challenge model, such as incorporating ASFV into feed/liquid for an oral and/or intranasal challenge post-vaccination, may be key to better understanding of the immune responses induced and obtaining protection following challenge. Therefore, to identify protective antigens needed for subunit vaccine development, there is a need to empirically define an appropriate ASFV challenge dose. This is important given that the correlates of protection are not yet available and the optimal antigen(s) for inducing protection have not yet been defined. Additionally, challenging animals immunized with a subunit vaccine candidate with a high dose of virulent ASFV that has been shown to work for evaluating efficacy of attenuated ASFV candidate vaccines may not be appropriate and hinder identification of antigen-specific immune responses that correlate with protection.

To date, the majority of ASFV immunization studies have used intramuscular administration of vaccine and the same route for challenge. Few studies aim to determine effective intranasal challenge doses of ASFV isolates that differ in virulence. The majority of immunization studies have used well-characterized domestic breeds, such as large white or landrace as the target animal for immunization studies (16, 27, 32, 67, 71, 74). To date, only a few groups have used indigenous breeds of pigs from ASFV endemic areas, such as Africa for vaccination research (83).

The high costs associated with BSL3 biocontainment laboratories and space constraints in such facilities have limited the number of challenge studies performed and hindered long-term monitoring of animals post-challenge. Studies have reported variable duration of monitoring post-challenge, ranging from 17 to 63 DPV and this does not provide consistent data for comparison of vaccine candidates (41, 64, 81). Thus, vaccine immunization and challenge protocols need to be standardized to allow uniform interpretation of outcomes.

## ASFV Candidate Vaccine-Induced Disease Exacerbation

Vaccinated pigs can potentially develop chronic ASF or severe pathology either post-vaccination and/or post-challenge. Following vaccination and challenge more severe clinical disease, when compared to the non-vaccinated animals, has been observed. Jancovich et al. (70) showed that vaccine-induced antibodies correlated with increased viremia. This observation was also supported by outcomes reported in several other studies (64, 70, 71). In the 1960s, live attenuated vaccines were used to immunize pigs following outbreaks of ASF in Portugal, Spain and Dominican Republic (53, 86). Although there were reports of survival and protection from naturally attenuated ASFV used, the biggest concerns with deploying LAVs is safety and the ensuing persistence of chronic forms of ASF in pig populations. Such persistence of chronic clinical signs were observed during evaluation of the attenuated ASFV NH/P68ΔA276R, which failed to confer protection against Arm07 challenge (32). In another study, pigs immunized with the ASFV-G-ΔL83L mutant had severe ASF clinical symptoms, similar to pigs inoculated with the parental ASFV-G virus, and either died from the infection or had to be euthanized (40).

The ASFV causes high mortality rates in domestic swine, regardless of gender and age (87). Another point to be considered is whether gender and sex differences have any effect on vaccination outcome (88). Netherton et al. (66) recently observed a variation in disease outcome between male and female immunized pigs. The authors reported that male immunized pigs showed enhanced ASF clinical disease, while female pigs had reduced viremia compared to control pigs (66).

## Conclusion and Future Perspectives

African swine fever virus causes acute hemorrhagic fever in pigs that results in high mortality and lack of a vaccine limits control to test and mass slaughter of infected and in-contact pigs. Sequencing genomes of attenuated and virulent strains, and targeted gene deletions from virulent strains have revealed genes encoding some of the factors involved in virulence and immune evasion, and with increasing spread of the disease, there is an impetus to sequence genomes of more isolates to identify relevant genes. It is clear that effective immunity depends on both antibody and cellular immune responses. Pigs immunized with naturally low virulence isolates or attenuated viruses produced by targeted gene deletions can induce protection against challenge by wild type virulent viruses. Virus antigens that are potential targets for inducing neutralizing antibodies have been identified and immunization with some of these antigens has been shown to confer partial protection. However, antigens that can elicit protective immunity, especially CD8^+^ T cell targets, have yet to be identified. Although several live attenuated ASFV are currently the most promising vaccine candidates, further work is needed to address some limitations, in particular scale up, prior to approval for deployment. Importantly, definition of correlates of protection against ASFV will enable rational identification of protective antigens for development of DIVA subunit vaccine. Recent studies have sequenced the warthog (*Phacocherus africanus*) and bush pig (*Potamochoerus larvatus*) genomes to better understand mechanisms of tolerance to ASFV infection, and how the disease burden is reduced in these swine species compare to domestic swine (89). This data will support current and future vaccine development strategies by comparing susceptible to resistant pig species.

## Author Contributions

WM, SL, RB, and SW planned and wrote the paper. HS, GM, and NS conducted literature review and wrote the paper.

### Conflict of Interest

The authors declare that the research was conducted in the absence of any commercial or financial relationships that could be construed as a potential conflict of interest.
